# The effect of hemolysis on quality control metrics for noninvasive prenatal testing

**DOI:** 10.1186/s12920-022-01280-2

**Published:** 2022-06-04

**Authors:** Yaya Guo, Dandan Yu, Kaisu Zhou, Jie Wang, Dongzhu Lei, Zhenpeng Xu, Weijiang Tang, Miaofeng Wu, Xingxing Fang, Jiankun Shen, Zhiyu Peng, Jiale Xiang

**Affiliations:** 1 BGI College, Zhengzhou University, Zhengzhou, 450007 China; 2 Henan Institute of Medical and Pharmaceutical Sciences, Zhengzhou University, Zhengzhou, 450052 China; 3 BGI Genomics, BGI-Shenzhen, Shenzhen, 518083 China; 4 Department of Obstetrics, Shenzhen Second People’s Hospital, Shenzhen, 518000 China; 5 Department of Genetics, Inner Mongolia Maternity and Child Health Care Hospital, Hohhot, 010020 China; 6 Center of Prenatal Diagnosis, Chenzhou No.1 People’s Hospital, Chenzhou, 423000 China; 7 BGI-Wuhan Clinical Laboratories, BGI-Shenzhen, Wuhan, 430074 China; 8 Clinical Laboratory of BGI Health, BGI-Shenzhen, Shenzhen, 518083 China; 9 College of Life Sciences, University of Chinese Academy of Sciences, Beijing, 100049 China

**Keywords:** cfDNA, Hemolysis, Hemoglobin, Noninvasive prenatal testing, Quality control metrics

## Abstract

**Background:**

Noninvasive prenatal testing (NIPT) is the testing of blood samples from pregnant women to screen for fetal risk of chromosomal disorders. Even though in vitro hemolysis of blood specimens is common in clinical laboratories, its influence on NIPT has not been well investigated.

**Methods:**

Peripheral blood samples were collected from 205 pregnant women and categorized according to the concentration of free hemoglobin in the plasma. After performing NIPT using massively parallel sequencing, the quality control metrics were analyzed and compared with samples that did not undergo hemolysis or samples redrawn from the same women.

**Results:**

The specimens were divided into four groups based on the concentration of free hemoglobin: Group I (0–1 g/L, n = 53), Group II (1–2 g/L, n = 97), Group III (2–4 g/L, n = 30), and Group IV (> 4 g/L, n = 25). There was no significant difference in the quality control metrics of clinical samples with slight or moderate hemolysis (Group II and III). However, samples with severe hemolysis (Group IV) showed a significantly increased rate of duplicated reads (duplication rate) and fetal fraction, as well as decreased library concentration compared with samples without hemolysis. Moreover, the increase in fetal fraction caused by hemolysis was confirmed by redrawing blood samples in Group IV.

**Conclusion:**

For NIPT using massively parallel sequencing, samples with slight or moderate hemolysis (≤ 4 g/L) are acceptable. However, careful consideration should be taken regarding the use of severely hemolyzed samples (> 4 g/L), since they might increase the risk of test failure.

**Supplementary Information:**

The online version contains supplementary material available at 10.1186/s12920-022-01280-2.

## Introduction

NIPT screens cell-free fetal DNA (cffDNA) fragments for common fetal aneuploidies, including trisomy 21 (T21), trisomy 18 (T18), and trisomy 13 (T13) [1, 2]. In recent years, it was expanded to rare autosomal aneuploidies, sex chromosome aneuploidies, and chromosomal microdeletions and microduplications [3]. cffDNA refers to extracellular DNA released from the placenta into maternal circulation, and it accounts for about 4–30% of total cell free-DNA (cfDNA) in maternal peripheral blood. The concentration of cffDNA, called the fetal fraction, increases with gestation time [4–6]. The fetal fraction is affected by maternal age, body mass index, medication exposure, race, and any aneuploidies, as well as fetal or maternal mosaicism and whether the gestation is a singleton or multiple fetuses [7–9]. Library construction and sequencing are two critical steps in NIPT. The library concentration, fetal fraction, duplication rate, mapping rate, and GC content are generally considered quality control metrics. These metrics strongly predict the reliability of NIPT results.

Hemolysis is the destruction of red blood cells, which leads to the release of hemoglobin and other intracellular components into the blood [10]. Hemolyzed specimens have red-colored plasma after centrifugation. Hemoglobin is a metalloprotein in red blood cells that accounts for about 96% of their dry content (by weight), and the free hemoglobin concentration can be used as an indicator of red blood cell destruction [11, 12].

In clinical laboratories, as much as 3.3% of blood specimens show various degrees of hemolysis [13]. The hemolysis is attributed to various causes, including maternal factors, puncture difficulties, improper operation, or improper storage during transport and separation. Importantly, hemolysis causes 40–70% of specimen nonconformities in clinical laboratories [13, 14]. For instance, it interferes with the evaluation of the concentration of several other blood constituents, such as meaningful variation in potassium and total bilirubin [15], as well as hematological testing analyses [16]. Hemolyzed samples are thus routinely rejected by most clinical and research laboratories [17, 18].

The influence of hemolysis on NIPT has not been well investigated. A recent study showed that hemolysis in Cell-Free DNA Collection Tubes had no impact on the fetal fraction in NIPT [19]. One of the limitations of this study was that the hemoglobin concentration was assessed visually rather than with automated spectrophotometric instrumentation. In addition, other quality control metrics, such as library concentration, duplication rate, mapping rate, and GC content, were not evaluated. Most importantly, the NIPT assay was based on a microarray; that is, there has not yet been an investigation of the impact of hemoglobin on NIPT using massively parallel sequencing, which is one of the most common NIPT techniques used worldwide.

In this study, we used clinical specimens to comprehensively investigate the effect of hemolysis on the quality control metrics of NIPT using massively parallel sequencing, including fetal fraction, library concentration, duplication rate, mapping rate, and GC content. Our results can be used by clinical laboratories to evaluate samples on reception and determine which should be rejected.

## Materials and methods

### Study design

This study was conducted in two parts. In the first part, we collected specimens from clinical laboratories of the Beijing Genomics Institute (BGI) from June 2020 to December 2021. The specimens were collected from pregnant women who received a NIPT test in a clinical setting. Specimens were categorized into four groups based on their free hemoglobin concentration. The effect of hemolysis was evaluated based on five quality control metrics: library concentration, fetal fraction, GC content, mapping rate, and duplication rate. The library concentration was used to assess the quality of the library, while the other four metrics were used to assess the sequencing quality. Duplication rate was defined as the proportion of reads of duplicate copies with the same barcode and the same 5' and 3' coordinates, reflecting the complexity of libraries [20]. Mapping rate was defined as the percentage of all input reads that were uniquely mapped. A low mapping rate indicated problems with library preparations or data processing [21].

In the second part, a new tube of blood was collected from several women whose specimens had been severe hemolyzed in the first part as soon as possible. The average duration between blood draw was 8 days. The samples were delivered to clinical laboratories for testing under the same storage and transportation conditions used earlier. This study was approved by the Institutional Review Board of BGI (No. BGI-IRB 21,007). All methods were carried out in accordance with its guidelines and regulations.

### Sample collection and processing

To investigate the effect of hemolysis, we prospectively collected hemolyzed specimens sent to BGI’s clinical laboratories for a NIPT assay. The degree of hemolysis was firstly evaluated by visual inspections. The collection of moderate and severe hemolyzed specimens were consecutive through the period of this study. Since the number of specimens with mild hemolysis was overly exceeded, the collection was randomized. Once the specimens were recruited, the concentration of free hemoglobin were measured by a hematology analyzer.

More specifically, 5 mL of maternal blood was collected from singleton pregnancies in Cell-Free DNA Collection Tubes (Geenseek, Guangzhou, China) and transported to the clinical laboratory (BGI, Shenzhen) within 4 days. The plasma was collected after centrifugation at 3900 × g for 15 min at 4 °C.

### Measurement of hemoglobin concentration

The free hemoglobin concentration of the specimens was determined with a Sysmex XN-9000 analyzer (Sysmex Corporation, Kobe, Japan) by photometry at 555 nm wavelength to minimize interference from turbidity and other sources [22]. This method utilized a sodium lauryl sulfate reagent [23], which forms a stable complex with oxidized hemoglobin.

### cfDNA extraction and library construction

cfDNA was extracted and enriched from 200 µL of plasma using a DNA Extraction Kit (BGI Biotech, Wuhan, China). According to the manufacturer’s instructions, cfDNA was collected on magnetic beads followed by washing and purification. Size selection was then performed to enrich the fetal fraction by discarding long fragments and collecting cfDNA fragments of the desired size range in two selection steps using different concentrations of carboxyl-beads.

After cfDNA extraction and enrichment, library construction was conducted using the Fetal Chromosome Aneuploidies (T21, T18, and T13) Detection Kit-Combinatorial Probe-Anchor Synthesis Sequencing Method (BGI Biotech, Wuhan, China) according to the manufacturer's instructions. In brief, end repair was performed with repair enzymes at 37 °C for 10 min and 65 °C for 15 min, followed by adaptor ligation with adaptors and ligase at 23 °C for 20 min. After purification, PCR was used to amplify cfDNA fragments that successfully ligated to the adaptors. These PCR products were purified and quantified using an ExKubit dsDNA HS Kit (Excell Bio, China).

The size distribution of the libraries was analyzed using an Agilent Bioanalyzer 2100 (Agilent Technologies, USA). Libraries with a concentration above 2 ng/µL were then pooled together. After pooling, denaturation and cyclization were carried out to generate single-stranded DNA circles, which would then form DNA Nanoballs (DNBs) by rolling circle replication (RCR). These DNBs were then quantified (8–40 ng/µL) and loaded onto sequencing chips, followed by sequencing on the MGISEQ-2000 sequencing platform (MGI, Shenzhen, China) [24, 25].

### Sequencing quality control and mapping

The libraries were sequenced on an MGISEQ-2000 sequencer (MGI, Shenzhen, China) using paired-end 35 bp reads [26]. The sequencing reads were mapped to the human reference genome (hg19) using Bowtie and SAMtools to create BAM and index files [27]. To calculate the fetal fraction, different methods were adopted for pregnancy with male or female fetuses. For a male fetus, the fetal fraction could be calculated by the proportion of reads mapping to the Y chromosome relative to those mapping to the whole genome. The fetal fraction in pregnancies with female fetuses was estimated using the FF-QuantSC algorithm previously developed by BGI [25, 28].

### Statistical analysis

The quality control metrics in each group were reported as mean values. They were considered to be scale variables in the analyses. First, the difference between the four groups was tested using the Mann–Whitney U test. To investigate the correlation between hemolysis and quality control metrics, Spearman's correlation coefficient was calculated. Next, the difference between samples with and without hemolysis was tested using a paired samples t-Test. A *p* value of less than 0.05 was considered statistically significant. Statistical analyses were performed with IBM SPSS Statistics, version 26.

## Results

### Characteristics of clinical samples

From June 2020 to December 2021, a total of 205 clinical blood specimens with different degrees of hemolysis were collected from BGI clinical laboratories. All samples were de-identified. The specimens were categorized into four groups according to their free hemoglobin concentration: Group I (n = 53), Group II (n = 97), Group III (n = 30), and Group IV (n = 25), with a concentration range of [0–1], (1–2], (2–4], and (4, ∞), respectively. Based on a visual assessment of plasma color, Group I samples were considered non-hemolyzed, whereas those of Group II, Group III, and Group IV were recorded as mild, moderate, and severely hemolyzed samples.

The mean maternal age in the groups was 30, 30, 30, and 31 years and the gestational week was 16^+1^, 16^+0^, 15^+4^, and 16^+2^ in Group I, Group II, Group III, and Group IV, respectively. There was no significant difference in the maternal age and gestational week of individuals in Groups II, III, or IV compared with Group I (Table 1).

**Table 1:** The characteristics and classification of clinical specimens

Classification	Visual inspection	Number	Hemoglobin level (g/L)	Maternal age in years (range)	Gestational week (range)	*P1*	*P2*
Group I		53	[0, 1]	30 (19–45)	16^+1^ (12^+0^–28^+0^)	–	–
Group II		97	(1, 2]	30 (19–50)	16^+0^ (12^+1^–27^+3^)	0.972	0.623
Group III		30	(2, 4]	30 (19–39)	15^+4^ (12^+0^–22^+1^)	0.904	0.541
Group IV		25	> 4	31 (22–41)	16^+2^ (12^+0^–19^+6^)	0.391	0.517

*P*1, the comparison of maternal age to group I. *P*2, the comparison of gestational weeks to group I

### Influence of hemolysis on the quality control metrics for NIPT

#### Between-group analysis

To examine the effect of hemolysis on the quality control metrics, NIPT using massively parallel sequencing was carried out on the 205 samples using an MGISEQ-2000 sequencer.

Library concentration is the primary quality control metric in NIPT. It is used to assess whether a library is suitable for sequencing. Based on our in-house experience, the concentration should be higher than 2 ng/μL to ensure a good quality sequence. The library concentration of samples in Group I, Group II, and Group III were all higher than 2 ng/μL. However, four samples (4/25, 16%) in Group IV failed to meet this threshold. These samples were processed a second time. The mean library concentration of Group I, Group II, Group III, and Group IV was 9.69 ng/μL, 11.25 ng/μL, 10.94 ng/μL, and 7.30 ng/μL, respectively. Overall, the library concentration of Group IV was significantly lower compared with Group I (*p* = *0.036*, Table 2).

**Table 2:** Comparison of the quality control metrics of four groups with different level of hemolysis

Quality control metric	Group I	Group II	Group III	Group IV
Library concentration (ng/μL)	9.69	11.25 *P* = *0.218*	10.94 *P* = *0.302*	7.30 *P* = *0.036*
Fetal fraction (%)	16.45	15.71 *P* = *0.382*	15.66 *P* = *0.694*	21.13 *P* = *0.002*
GC content (%)	40.69	40.70 *P* = *0.840*	40.68 *P* = *0.947*	40.68 *P* = *0.768*
Mapping rate (%)	75.96	75.96 *P* = 0.976	75.87 *P* = 0.448	75.79 *P* = *0.728*
Duplication rate (%)	9.40	8.49 *P* = *0.187*	7.89 *P* = *0.132*	12.91 *P* = *0.034*

*P* value, the comparison of each metric to group I

The other four quality control metrics, fetal fraction, GC content, mapping rate, and duplication rate, were obtained from the sequencing data. There was no significant difference between the four groups in GC content or mapping rate (Table 2). However, the fetal fraction was significantly higher in Group IV than in Group I (21.13 vs. 16.45%, *p* = *0.002*), and the duplication rate was also significantly higher in Group IV than Group I (12.91 vs. 9.40%, *p* = *0.034*).

**Fig. 1:**
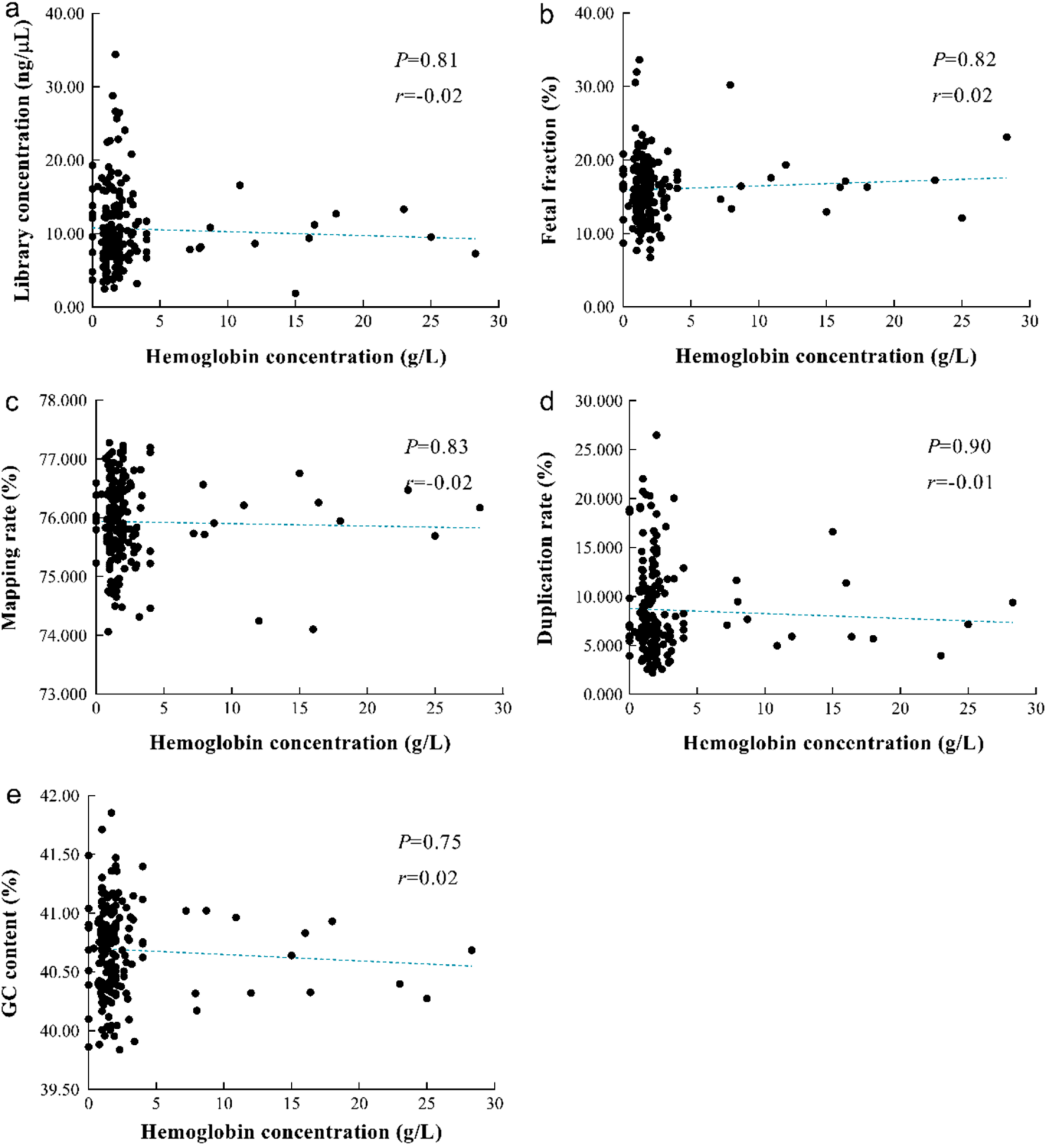
The correlation analysis of hemolysis and quality control metrics. (**a**–**e**) The analysis of correlation between hemoglobin concentration and (**a**) library concentration, (**b**) fetal fraction, (**c**) mapping rate, (**d**) duplication rate as well as (**e**) GC content

**Fig. 2:**
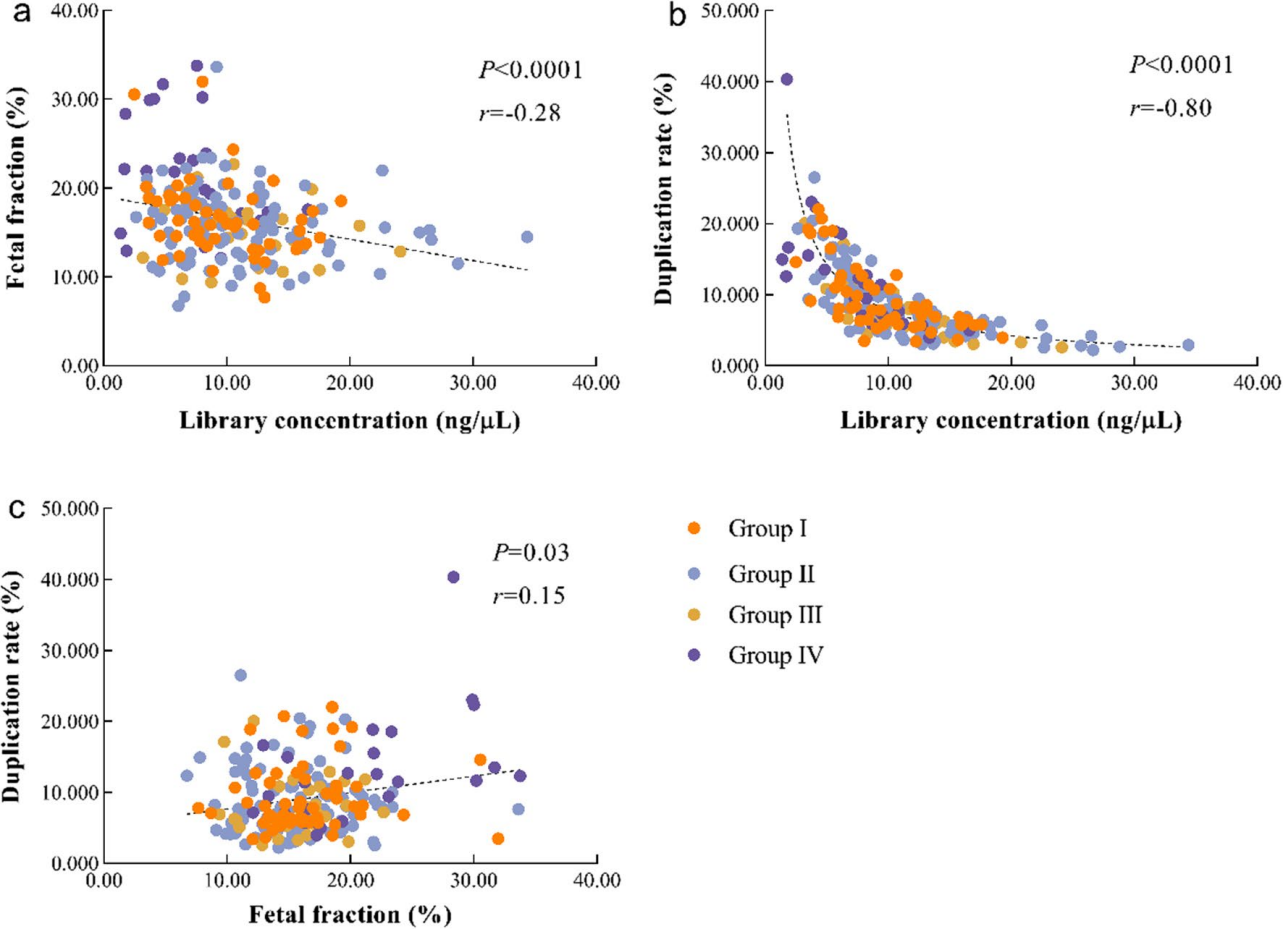
The correlations between different quality control metrics. (**a**–**c**) The analysis of correlation between (**a**) library concentration and fetal fraction, (**b**) library concentration and dupliation rate,  (**c**) fetal fraction and duplication rate

To further investigate the correlation between hemolysis and the quality control metrics, we calculated the Spearman's correlation coefficient (Fig. 1). It was apparent that the level of hemolysis was not correlated with the quality control metrics (Fig. 1a–e). However, the library concentration was negatively correlated with the duplication rate (Fig. 2b). There was no correlation between fetal fraction and library concentration (Fig. 2a) or duplication rate (Fig. 2c).

#### Within-group analysis

Of the 25 patients whose samples had severe hemolysis (Group IV), 21 agreed to come back to redraw blood. The new samples were collected within an average of eight days (3–12 days) after the first blood collection. All samples did not hemolyze at the second blood draw. We were thus able to use pairwise comparison to compare the quality control metrics of samples from the same woman with and without hemolysis.

The mean library concentration was 14.24 ng/μL for the non-hemolyzed samples, which was significantly higher than the hemolyzed specimens (8.11 ng/μL, *p* < *0.001*, Table 3). The duplication rate was also significantly reduced (6.84 vs. 12.64%, *p* = *0.007*). As in the between-group analysis, there was no change in the GC content or mapping rate. Interestingly, the fetal fraction was higher in the hemolyzed specimens, although they were collected an average of eight days earlier (20.70 vs. 16.18%, *p* = *0.006*).

**Table 3:** The comparison of hemolyzed and non-hemolyzed specimens from the same patients

Quality control metric	Hemolyzed specimen (n = 21)	Non-hemolyzed specimen (n = 21)	*P*
Library concentration (ng/μL)	8.11	14.24	< 0.001
Fetal fraction (%)	20.70	16.18	0.006
GC content (%)	40.68	40.80	0.162
Mapping rate (%)	75.69	75.61	0.800
Duplication rate (%)	12.64	6.84	0.007

## Discussion

Hemolysis is one of the leading causes of specimen rejection in clinical laboratories [29]. Although the prevalence varies with geographical area, transportation, or laboratory setting, it can be as high as 3.3%. To date, the impact of hemolysis on NIPT using massively parallel sequencing is not known. In this study, we quantified the free hemoglobin concentration in plasma as an indicator of the degree of hemolysis. Through between-group and within-group analyses, we demonstrated that mild or moderate hemolysis had no impact on the quality control metrics for NIPT using massively parallel sequencing, whereas severe hemolysis significantly affected the library concentration, fetal fraction, and duplication rate. This is the first study to investigate the effect of hemolysis on the quality control metrics for NIPT via massively parallel sequencing. Our data provided evidence to support clinical and research laboratories as they decide whether to accept or reject samples.

Library concentration is the primary quality control metric for NIPT. It was apparent that severe hemolysis resulted in a low library concentration. This could be explained by hemoglobin inhibiting DNA polymerase activity [30]. To construct sequencing libraries for NIPT using massively parallel sequencing, PCR amplification was necessary because the cfDNA extracted from 200 µL of plasma was not enough for sequencing. In addition, we found that the library concentration correlated with duplication rate, which has also been shown in other next-generation sequencing studies [31, 32]. This was because the inhibited amplification reduced the complexity of the library, leading to a higher duplication rate [33]. This reinforces the fact that libraries with a low concentration should not be used for sequencing.

Our data showed that the fetal fraction was significantly higher in samples with severe hemolysis. It is well established that fetal fraction increases with gestational time [34]. In the between-group analysis, there was no difference in the average gestational week of the four groups. However, the fetal fraction was significantly higher in Group IV.

The within-group analysis examined samples redrawn from women whose first samples were severely hemolyzed. In theory, the fetal fraction should have been higher in the second samples, since they were drawn an average of eight days later. However, it was significantly lower; in other words, there was a higher proportion of fetal DNA in the earlier, severely hemolyzed samples. This might be partially attributable to lysis of fetal nucleated red blood cells (NRBCs) during in vitro hemolysis, which would release fetal genomic DNA and thus increase the fetal fraction in maternal peripheral blood. This hypothesis was supported by a previous study, which demonstrated that most NRBCs in maternal peripheral blood before 24 weeks of gestation were of fetal origin, and after that the majority were of maternal origin [35]. Since the average gestation age in our study was approximately 16 weeks, we hypothesized that at that period more fetal NRBCs than maternal cells burst, thus increasing the fetal fraction. However, this hypothesis awaits verification by further investigation.

The mapping rate and GC content were not affected in hemolyzed specimens. The mapping rate is used to evaluate the proportion of uniquely mapped reads out of all input reads. When the quality of the library is low, such as following contamination by other samples, the mapping rate is low. GC content is another critical metric. The average GC content in human genomes ranges from 35 to 60%, with a mean of 41% [36]. Variation of GC content can indicate bias in library construction.

One concern regarding hemolysis is the lysis of leukocytes, which releases maternal genomic DNA into the plasma. Contamination with maternal genomic DNA would introduce a great deal of background noise and interfere with NIPT analysis. To prevent the lysis of leukocytes, cfDNA collection tubes are generally used in clinical practice. cfDNA collection tubes used in this study contain preservation agents to stabilize white blood cells, inhibiting them from releasing DNA into the plasma. Thus, when hemolysis occurs, red blood cells rupture but leukocytes will not be simultaneously lysed [37, 38]. This is because red blood cells are less stable than white blood cells and are more likely to rupture [39].

One limitation of this study is the lack of sufficient samples with chromosomal abnormalities confirmed by amniocentesis. Such samples would facilitate the assessment of whether hemolysis affects the results of NIPT. During the period of this study, only two hemolyzed samples with confirmed prenatal chromosomal abnormalities—chromosome 22q11.2 deletion syndrome (DiGeorge syndrome)—were available. The deletion in one sample was 2.9 Mb; in the other, 1.5 Mb. The two samples had mild hemolysis, and all five quality control metrics passed the cutoffs. More importantly, NIPT successfully identified the two deletions (Additional file 1: Figure S1 and Additional file 2: Figure S2). All the other samples were screened negative for chromosomal abnormalities, thus no confirmation tests were performed. These data show that specimens with mild hemolysis should not be rejected and can be processed as usual.

In conclusion, samples with slight or moderate hemolysis (free hemoglobin concentration ≤ 4 g/L) are acceptable for NIPT using massively parallel sequencing because no impact on the quality control metrics was found. Unnecessary rejection of samples can thus be avoided by measuring the degrees of hemolysis. However, greater attention should be paid to severely hemolyzed samples (free hemoglobin concentration > 4 g/L) because severe hemolysis significantly decreased duplication rate, increased library concentration and fetal fraction. Accepting these specimens might increase the risk of test failure and thus waste time during prenatal testing.

These findings provide a scientific basis for NIPT laboratories to evaluate hemolyzed samples for high-throughput sequencing. Rejecting samples with free hemoglobin concentration > 4 g/L would reduce experimental errors which could harm patients and also lead to repeated specimen collection and analysis, increasing the cost of NIPT [40].

## Supplementary Information

**Additional file 1.** The CNV plot of NIPT and prenatal diagnosis of sub-chromosomal CNVs hemolyzed sample 1.

**Additional file 2.** The CNV plot of NIPT and prenatal diagnosis of sub-chromosomal CNVs hemolyzed sample 2.

## Data Availability

The data used and/or analyzed during the current study are available from the corresponding author on reasonable request. The data are not publicly available due to privacy or ethical restrictions.
