# Supplementary material for: The effect of hemolysis on quality control metrics for noninvasive prenatal testing
Source: BMC Med Genomics. 2022 Jun 4;15:125. doi: 10.1186/s12920-022-01280-2 (PMC9167518; doi:10.1186/s12920-022-01280-2)
Supplement: Supplementary file 1 — Additional file 1. The CNV plot of NIPT and prenatal diagnosis of sub-chromosomal CNVs hemolyzed sample 1. [file 12920_2022_1280_MOESM1_ESM.pdf]

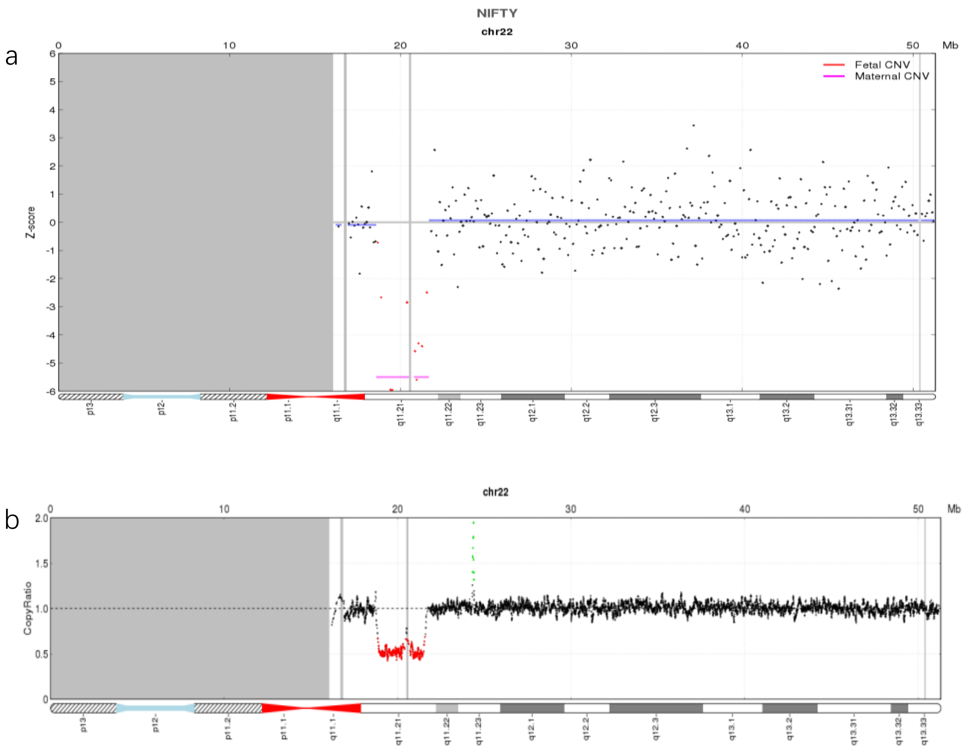

Figure S1 The CNV plot of NIPT and prenatal diagnosis of sub-chromosomal CNVs hemolyzed sample 1. a, the CNV plot result of NIPT is seq[GRCh37]del(22q11.21q11.21)chr22:g.18578067-21663666del. b, the CNV plot result of prenatal diagnosis is seq[GRCh37]del(22q11.21q11.21)chr22:g.18765311-21635978del. Red indicate chromosomal deletions region.
