# Supplementary material for: The effect of hemolysis on quality control metrics for noninvasive prenatal testing
Source: BMC Med Genomics. 2022 Jun 4;15:125. doi: 10.1186/s12920-022-01280-2 (PMC9167518; doi:10.1186/s12920-022-01280-2)
Supplement: Supplementary file 2 — Additional file 2. The CNV plot of NIPT and prenatal diagnosis of sub-chromosomal CNVs hemolyzed sample 2. [file 12920_2022_1280_MOESM2_ESM.pdf]

a

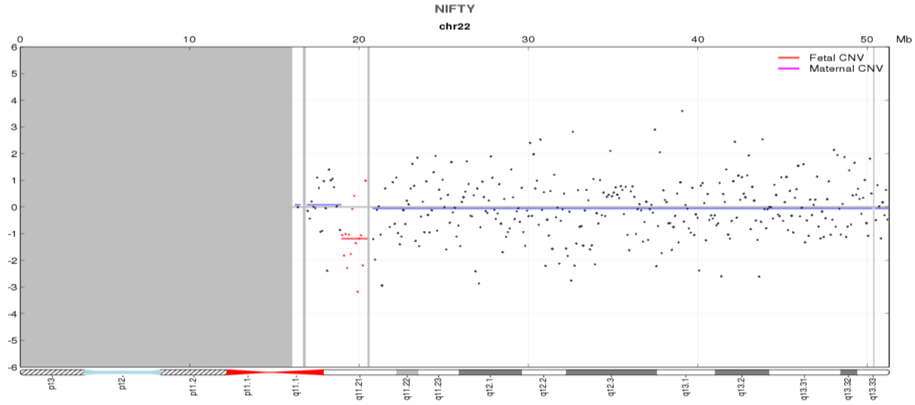

b

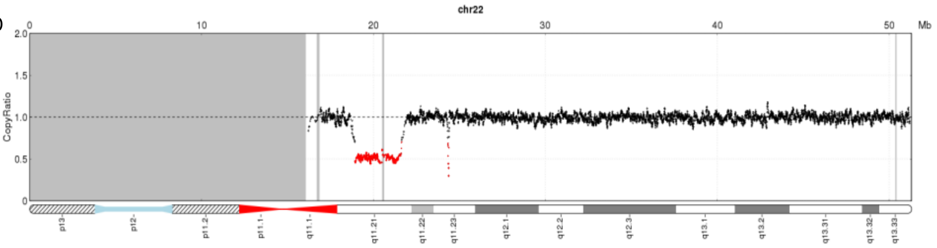

Figure S2 The CNV plot of NIPT and prenatal diagnosis of sub-chromosomal CNVs hemolyzed sample 2. a, the CNV plot result of NIPT is seq[GRCh37]del(22q11.21q11.21)chr22:g.18957712-20501581del. b, the CNV plot result of prenatal diagnosis is seq[GRCh37]del(22q11.21q11.21)chr22:g.18679614-21746118del.
